# Antimicrobial activities of commercial nanoparticles against an environmental soil microbe, *Pseudomonas putida *KT2440

**DOI:** 10.1186/1754-1611-3-9

**Published:** 2009-06-26

**Authors:** Priyanka Gajjar, Brian Pettee, David W Britt, Wenjie Huang, William P Johnson, Anne J Anderson

**Affiliations:** 1 Department of Biological Engineering, Utah State University, Logan Utah 84321, USA; 2 Department of Biology, Utah State University, Logan Utah 84321, USA; 3 Chemical Engineering, University of Utah, Salt Lake City, Utah 84112, USA; 4 Geology & Geophysics, University of Utah, Salt Lake City Utah 84112, USA

## Abstract

**Background:**

The release of heavy metal-containing nanoparticles (NP) into the environment may be harmful to the efficacy of beneficial microbes that function in element cycling, pollutant degradation and plant growth. Nanoparticles of Ag, CuO and ZnO are of interest as antimicrobials against pathogenic bacteria. We demonstrate here their antimicrobial activity against the beneficial soil microbe, *Pseudomonas putida *KT2440.

**Results:**

Toxicity was detected in a KT2440 construct possessing a plasmid bearing the *luxAB *reporter genes. "As manufactured" preparations of nano- Ag, -CuO and -ZnO caused rapid dose-dependent loss of light output in the biosensor. Cell death accompanied loss in Lux activity with treatments by nano-Ag and -CuO, but with -ZnO the treatments were bacteriostatic rather than bactericidal. Bulk equivalents of these products showed no inhibitory activity, indicating that particle size was determinant in activity. Flow Field-Flow Fractionation (FlFFF) of an aqueous suspension of the nano-CuO and ZnO revealed a small proportion of 5 nm NP and aggregated particulates with sizes ranging between 70 nm and 300 nm; the majority portion of material was aggregated into particles larger than 300 nm in size. Thus within the commercial preparation there may be microbially active and inactive forms.

**Conclusion:**

The "as-made" NP of Ag, CuO and ZnO have toxic effects on a beneficial soil microbe, leading to bactericidal or bacteriostatic effects depending on the NP employed. The lack of toxicity from bulk materials suggests that aggregation of the NP into larger particles, possibly by factors present in the environment may reduce their nontarget antimicrobial activity.

## Background

Nanotechnology has attracted global attention because nanoparticles (NP) have properties unique from their bulk equivalents. NP of Ag, CuO and ZnO are being used industrially for several purposes including amendments to textiles, cosmetics, sprays, plastics and paints [1]. A common feature of these three NP is their antimicrobial activity [2-8]. The antimicrobial activity of NP largely has been studied with human pathogenic bacteria, mainly *Escherichia coli *and *Staphylococcus aureus*. Nano-Ag is inhibitory to *E. coli *[5,9-16] and *S. aureus *[5,9,12,16]. These microbes also are sensitive to nano-CuO and nano-ZnO [17,18].

NP of Ag, CuO and ZnO are reported to attack bacterial membranes. Short exposure of *E. coli *cells to nano-Ag destabilizes the outer membrane, collapses the plasma membrane potential and decreases ATP [10]. Pits in *E. coli *cell walls were observed after nano-Ag treatment [13] and promoted release of green fluorescent protein from transformed *E. coli *cells [14]. Exposure of *E. coli *to nano-ZnO also causes loss in membrane integrity [6]. Likewise, toxicity of NP of CuO and ZnO are connected with cell membrane damage [17].

NP action may be due in part to their release of free ions. Heavy metal ions have diverse effects on bacterial cell function. For Cu ions, the mechanism may involve oxidative stress [8]. The redox cycling of Cu ions results in depletion of glutathione and affects the sulfhydryl groups of proteins causing DNA damage and lipid oxidation [7]. Like Cu, Zn also is an essential element for cells; levels of Zn above the essential threshold level inhibit bacterial enzymes including dehydrogenase [19] and certain protective enzymes, such as thiolperoxidase, and glutathione reductase [20]. Zn inhibition of NADH oxidase is proposed to impede the respiratory chain of *E. coli *[21]. Additionally, loss of membrane potential is associated with inhibition by Zn ions at cytochrome c oxidase in *Rhodobacter sphaeroides *[22]. Ag ions inactivate proteins with SH groups and prevent the ability of DNA to replicate [23]. Holt and Bard [24] propose that NADH dehydrogenase in the electron transport chain of *E coli *is inhibited by Ag ions.

Extensive use and increasing demand for NP will lead to their accumulation in the environment, especially in landfills and their water effluents. Control of pathogenic microbes by antimicrobial NP is a promising approach to defeat the multiresistant pathogens such as methicillin-resistant *S. aureus *[18]. However, nontarget effects on the populations of microbes that play beneficial roles in the environment could have negative consequences. Many microbes have essential roles in element cycling, (carbon, sulfur, nitrogen, etc.), while others degrade pollutants and promote plant growth [25-31]. Nowack and Bucheli [32] found little published information about the release of NP in the environment in their efforts to model the risk of Ag NP. Novel and unprecedented sources are likely: recently, commercially available nano-Ag-treated socks were found to release Ag upon washing the socks [33]. Concern for nontarget effects of environmental accumulation of Ag has been raised [34].

The toxicity of NP against environmental microbes has been little studied. *Vibrio fisheri *has been used because of its natural light emitting property in assessment of toxicity and *Bacillus subtilis *has been examined as an example of a spore-forming bacterium [4,5,17]. The aim of this study was to evaluate the antimicrobial activity of nano-Ag, nano-CuO and nano-ZnO using a biosensor constructed in *Pseudomonas putida *KT2440. This pseudomonad is beneficial in the environment because of its bioremediation potential and it is a strong root colonizer [25,35,36]. The biosensor was constructed to emit light from *luxAB *genes under the control of a promoter containing a single heavy metal binding domain (MTCGHC). Because the luciferase encoded by *luxAB *requires FMNH_2 _as a substrate, expression from this promoter permits light output dependent on the energy status of the cells [37].

We report on the responses of the biosensor to NP of Ag, CuO and ZnO in comparison with the effects of bulk equivalents and free metal ions. We examined how loss of Lux activity correlated with changes in culturability of the cell as an effort to understand more of the potential environmental impacts of NP, a need discussed by Nowack and Bucheli [32]. We also document the sizes of the NP in aqueous suspension of the nano-metal oxides through the use of Flow Field-Flow Fractionation (FlFFF); aggregation of commercial preparations of NP is commonly reported.

## Methods

### Chemicals

ATTOSTAT (NLC Laboratories, Salt Lake City, UT) was used as the nano-Ag source, with NP of a reported size 10 nm and a concentration of 30 mg Ag/L. The bulk Ag source was from Alfa Aesar, Ward Hill, MA, with a reported particle size of 44,000 nm. Bulk and NP of CuO and ZnO were purchased from Sigma-Aldrich, St. Louis, MO. The reported "as manufactured" sizes were: nano-CuO, 33 nm; nano-ZnO, 50–70 nm; bulk CuO, 8000–9000 nm; and ZnO, less than 1000 nm. Exposure to ions was from solutions of CuCl_2_, Zn(NO_3_)_2 _and AgNO_3_. All solutions were prepared in distilled, sterile water.

### Biosensor construction and use

The biosensor was constructed in strain *P. putida *KT2440 to harbor a plasmid with a *luxAB *fusion to a Cu-responsive promoter [Pettee *et al*., unpublished]. Oligonucleotide primers were designed to amplify approximately 500 bps 5' to 100 bps 3' downstream of the translational start site at locus PP_0588 in wild type *P. putida *KT2440. The primers were: For, CGATGCGGTATTTGTTGATCT and Rev, AATCGCAGTGAGGATCTGCT. PCR products containing the PP_0588 promoter region were ligated to the promoterless *luxAB*::*npt *cassette in plasmid pCR2.1 5' bearing resistance genes for kanamycin and ampicillin (Invitrogen.com) in *E. coli*. Determination of the promoter orientation in the clones was achieved by PCR analysis using a primer to the 5' end of the *luxA *gene in the reverse orientation and identifying PCR products when used with the 5' promoter primer of PP_0588, 5'-CGATGCGGTATTTGTTGATCT-3'. The *luxA *primer sequence was 5'-CAACCAAATTTTCCCCAAGA-3'. Positive clones were ultimately confirmed by the presence of Lux activity and ability to grow on kanamycin at 20 μg/ml. The PP_0588 *lux *fusion was removed from the pCR2.1 vector and inserted into the stable plasmid pCPP45, bearing a resistance gene for tetracycline, for triparental mating into *P. putida *KT2440.

The PP_0588 cells were stored in 15% glycerol at -80°C. Logarithmic phase cells were generated by reculturing from an overnight culture grown in minimal medium (MM) with shaking at 25°C to OD600_nm _= 0.1. MM contained in 1 L: 10.5 g K_2_HPO_4_, 4.5 g KH_2_PO_4_, 0.5 g sodium citrate (2H_2_O), 1.0 g (NH_4_)_2 _SO_4_, 0.25 g MgSO_4_.7H_2_O, and 2.0 g sucrose. The culture (200 ml) was centrifuged at 10,000 g for 10 min and the cells were resuspended in 200 ml sterile distilled water and used immediately in the Lux assay. After dividing into 50 ml aliquots in 125 ml flasks, the suspensions were treated with NP, bulk material or ions at defined final concentrations or were left without treatment as a control. Initially the cells were treated with 0.1, 1 and 10 mg metal (M)/L to determine the sensitivity range. Subsequently doses were adjusted to determine the level at which toxicity was observed. Flasks were shaken at 200 rpm and 25°C during the study. At defined times, 200 μl of the suspensions were transferred in triplicate into well plates for Lux readings. The luciferase substrate, 1% decanal in ethanol, 10 μl, was added automatically in the L MAXII Luminometer (Molecular Devices Corporation, Sunnyvale CA). Light output was recorded with a 10 sec. exposure. Generally samples were assayed every 10 minutes up to 1 h. At each sampling time, the Lux activity from three aliquots of the cell suspension was measured. Each treatment was replicated in three or more separate studies.

### Assessment of culturability

Cells, after a 60 minute treatment with or without metal exposure, were assayed for culturability by dilution plating on salt-free Luria Broth (Difco, Sparks, MD) agar medium. Colonies were counted after 24 h incubation at 28°C and the colony forming units (Cfu)/ml determined.

### Fractionation of nano-metal oxide particles

An aqueous suspension of 10,000 mg Cu/L of nano-CuO or nano-ZnO in sterile distilled water was filtered sequentially through sterile filters with pore sizes of 450 and 200 nm (Whatman Inc., Florham Park, NJ, USA). The filtrates were collected and diluted 5, 10, 100 or 1000-fold into cultures of KT2440 to determine effect on light output as described above. After 60 minutes of exposure cells were plated to determine culturability.

### Flow Field-Flow Fractionation (FlFFF) and ICP-MS analysis

Suspensions of "as manufactured" nano-CuO and ZnO were fractionated according to size using asymmetric FlFFF (AF4) (Postnova Analytics, Landsberg, Germany). The operational procedure for FlFFF followed published procedures [38-40]. In FlFFF, carrier fluid (and introduced sample) flowed down the length of a channel bounded along its length by a membrane. NP size separation occurred in the presence of a cross-flow field perpendicular to the flow axis, in which the particles migrated differentially across the channel and aligned themselves within different streamlines in the laminar parabolic flow field, resulting in different-sized particles being carried toward the channel exit at different velocities. Under the FlFFF conditions used as described in Table 1, the elution time of a NP was proportional to its size; two experimental conditions were used to best tune the instrument to particle size of interest. Operating condition I (Table 1) was used for fractionation in the size range between 10 to 250 nm, and was calibrated using colloidal gold and fluorescent latex beads with known sizes (10, 98 and 200 nm, respectively). Operating condition II (Table 1) was used for fractionating particles smaller than 10 nm, and was calibrated using colloidal silver and colloidal gold with known sizes (5 nm and 10 nm). NPs with high diffusivity (small size) relative to the applied cross flow were eluted in the so-called "void peak". Particles with high fluid drag (large size) relative to diffusivity were held against the membrane until elution in the so-called "rinse peak" upon relaxation of the cross flow.

**Table 1:** Operation conditions for separation of particles of different size by FlFF

Operating conditions for particles 10–250 nm						
Focus step	Elution time min	Flow rate Tip ml/min	Flow rate Focus ml/min	Flow rate Cross ml/min	Flow rate Detector ml/min	Flow rate Slot ml/min
Elution step	NA	0.1	3.9	1	0.4	2.6
Stage 1	7	4	NA	1	0.4	2.6
Stage 2	5	3.7	NA	0.7	0.4	2.6
Stage 3	5	3.7	NA	0.7	0.4	2.6
Rinse step	5	3	0	0	0.4	2.6
						
Operating conditions for particles less than 10 nm						

Focus step	Elution time min	Flow rate Tip ml/min	Flow rate Focus ml/min	Flow rate Cross ml/min	Flow rate Detector ml/min	Flow rate Slot ml/min
Elution step	NA	0.1	4.4	3.5	0.4	0.6
Stage 1	12	4.5	NA	3.5	0.4	0.6
Stage 2	0.5	3.0	NA	2	0.4	0.6
Stage 3	0.5	2.0	NA	1	0.4	0.6
Rinse step	8	3	0	0	0.2	2.8

The injection flow rate was 0.1 ml/min, the time of injection 5 min and the time for transit of sample 1 min for both separation conditions. NA, not applicable.

The dimensions of the asymmetric FlFFF channel used were 27.3 cm in length, 224 μm in thickness. The channel volume was 0.71 ml, calculated according to described methods [41]. The membrane used was made from regenerated cellulose (10 K Dalton pore size). Milli-Q water (Millipore System) with 2% v/v FL-70 was used as carrier. The pH of the carrier solution was 8.93. Three detectors (UV absorbance, fluorescence and inductively coupled plasma mass spectrometry [ICP-MS]) were used in series downstream of the FlFFF to analyze the fractionated sample. FlFFF coupled with ICP-MS allowed online simultaneous determination of particle size distribution and elemental distribution of the nanomaterials [40]. The ICP-MS Agilent 7500 ce (Agilent Technologies Inc., Santa Claram, CA, USA) was used in continuous mode. The general operating parameters are summarized in Table 2.

**Table 2:** Description of operational conditions for ICP-MS of fractionated materials.

**OPERATIONAL CONDITIONS**	
RF power (W)	1550

Plasma gas flow rate (L/min)	15

Hydrogen flow rate (mL/min)	2.5

Helium flow rate (mL/min)	2.5

Carrier flow rate (L/min)	0.9

Make-up gas (L/min)	0.1

Auxiliary gas (L/min)	0.9

Sample flow rate (mL/min)	0.4

Acquisition time per isotope (sec)	0.1

Repetition	3

Total acquisition time for 19 isotopes (sec)	2.85

Total running time (sec)	1880

Tuning solution:	
% RSD	< 3%

Sample nebulizer tubing:	
Material	Tygon
Internal diameter (mm)	1.02

AF4 carrier tubing:	
Material	PEEK
Internal diameter (mm)	0.25

## Results

### Exposure to nano-Ag, bulk-Ag and Ag ions

**Figure 1:**
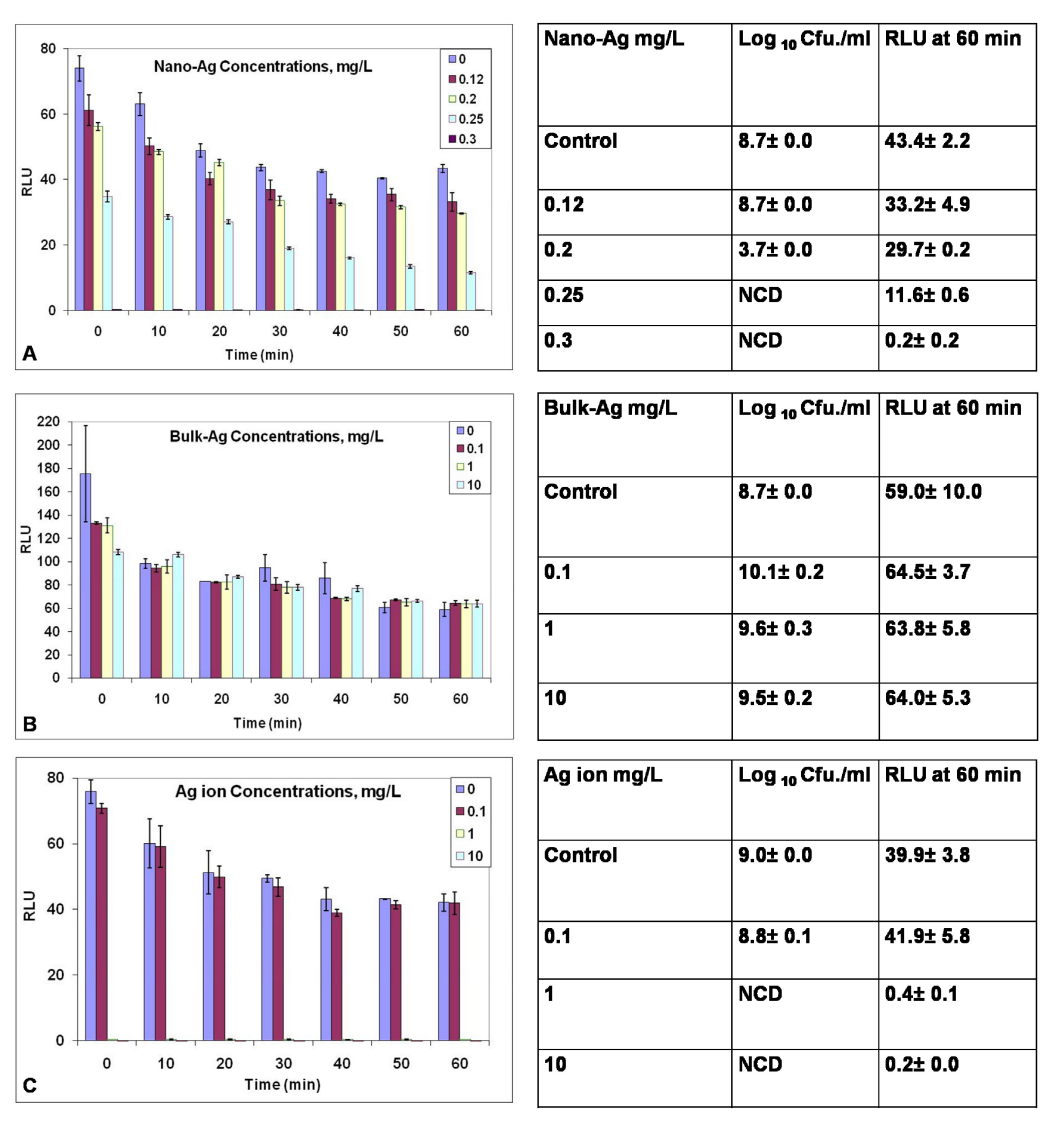
**Response of the *P putida *KT2440 biosensor to nano-Ag (A), bulk Ag (B) and Ag ions (C) at defined doses of mg Ag/L**. Changes in Lux output (Relative light units RLU) and cell culturability (colony forming units Cfu) are shown. Studies were performed as described in Methods. NCD = No culturable cells detected. Data are from one study typical of at least three generated under the same conditions. Means and standard errors are shown.

As shown in Fig. 1, nano-Ag was toxic to the biosensor. Treatments above 0.2 mg Ag/L caused immediate loss in light output (Fig. 1A). When treated with bulk Ag, no loss in Lux output was observed even for concentrations of 10 mg Ag/L (Fig. 1B). Cells also exhibited loss in light output with treatment from Ag ions with 1 and 10 mg/L doses (Fig. 1C). Treatment with a range of lower doses showed that 0.2 Ag ion/L also caused rapid loss in light output (data not shown). Consequently, there was a sharp threshold between 0.1 and 0.2 mg/L for toxicity with the Ag ions.

The tables adjacent to the RLU graphs report the changes in culturability of the cells transferred to plating medium after 60 minutes of treatment compared with unchallenged controls. For treatment with nano-Ag and Ag ions, loss of Lux activity correlated with loss in culturability. No loss in Lux output or culturability was observed with exposure to bulk Ag. At 0.25 mg/L nano-Ag no culturable cells were obtained; with Ag ion a culturability threshold near 0.2 mg Ag ion/L (data not shown) was determined.

### Exposure to nano-CuO, bulk-CuO and Cu ions

**Figure 2:**
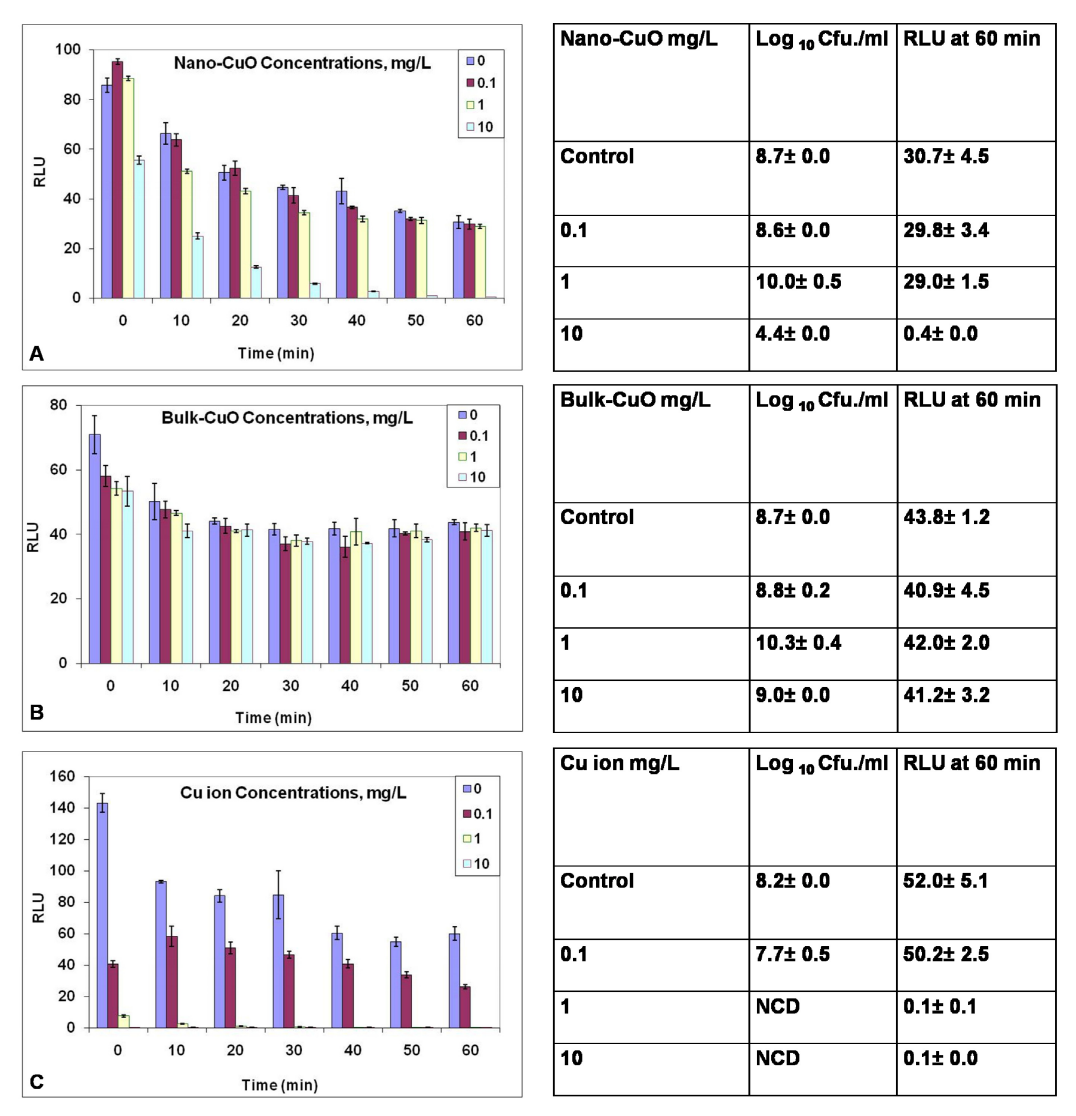
**Response of the *P putida *KT2440 biosensor to nano-CuO (A), bulk CuO (B) and Cu ions (C) at defined doses of mg Cu/L**. Changes in Lux output (Relative light units RLU) and cell culturability (colony forming units Cfu) are shown. Studies were performed as described in Methods. NCD = No culturable cells detected. Data are from one study typical of at least three generated under the same conditions. Means and standard errors are shown.

The biosensor also showed loss in Lux activity when treated with nano-CuO and Cu ions but not with bulk CuO. Fig. 2A demonstrates that treatment with 10 mg Cu/L from the nano-CuO caused a time-dependent loss in light output whereas bulk CuO was inactive (Fig. 2B). Treatment with 1.0 and 0.1 mg Cu/L nano-CuO caused no effect (Fig. 2A). Ten mg Cu/L nano-CuO rapidly reduced RLU, and a toxicity threshold showing rapid RLU reduction was observed between 5 mg and 7 mg Cu/L (data not shown). Toxicity of the Cu ion, from CuCl_2_, towards the biosensor was apparent at 1.0 mg Cu/L with 0.1 mg/L having little effect (Fig. 2C). A rapid RLU reduction was observed for 0.5 mg/L (data not shown). Thus, nano-CuO was about ten-fold less active than the free ions for the biosensor response. To confirm that toxicity with the Cu^2+ ^was due to the metal ions rather than the Cl^-^, the biosensor was exposed to Cl^- ^from NaCl. At a dose level where Cl^- ^was at the same concentration as that from CuCl_2 _when Cu was present at 10 mg/L there was no observed toxicity (data not shown).

Loss in Lux correlated with loss in culturability upon exposure to Cu ions and reduced culturability upon exposure to nano-CuO at 10 mg/L treatment. Cell culturability did not decline with bulk CuO exposure.

### Exposure to nano-ZnO, bulk-ZnO and Zn ions

**Figure 3:**
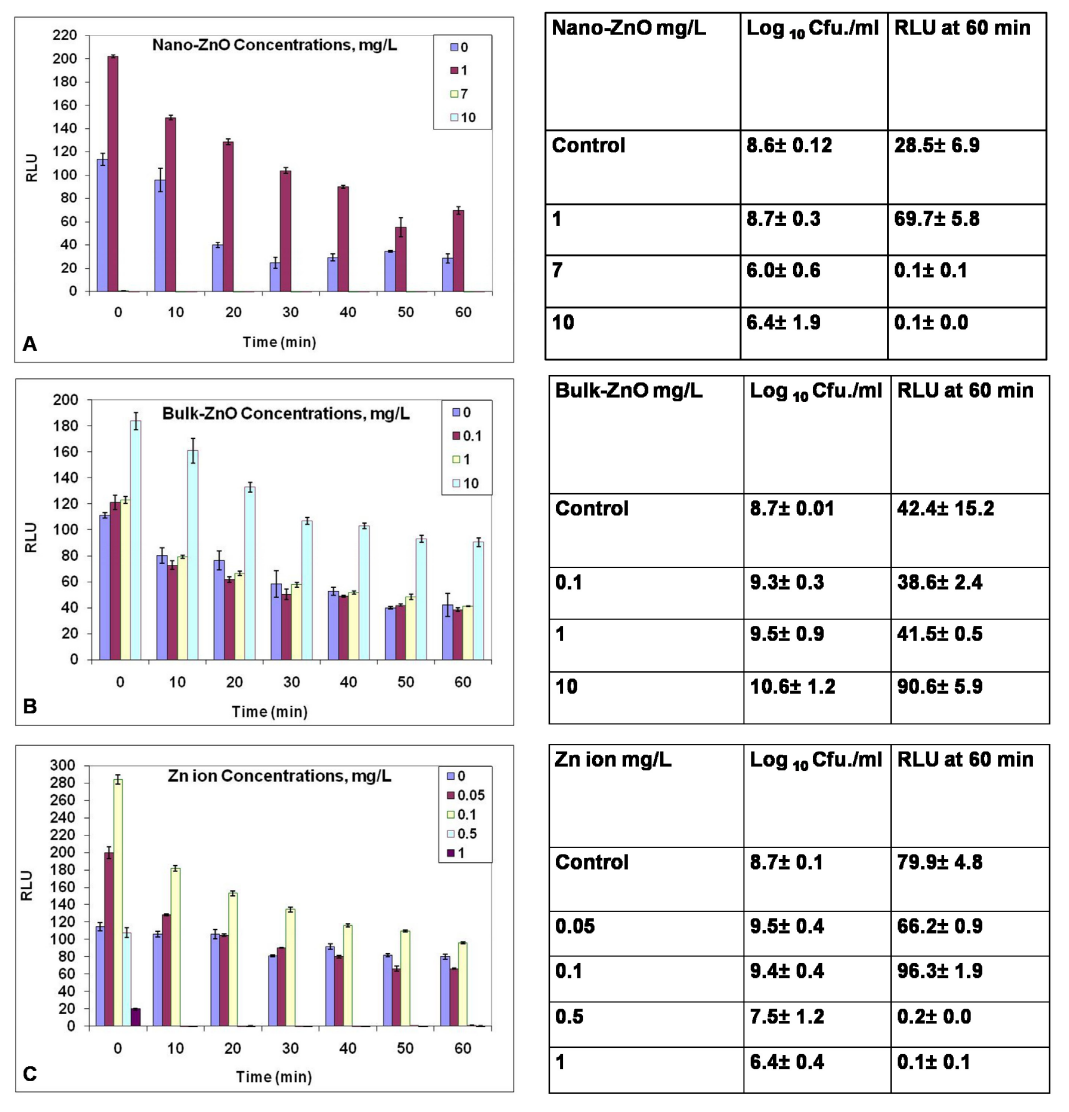
**Response of the *P putida *KT2440 biosensor to nano-ZnO (A), bulk ZnO (B) and Zn ions (C) at defined doses of mg Zn/L**. Changes in Lux output (Relative light units RLU) and cell culturability (colony forming units Cfu) are shown. Studies were performed as described in Methods. Data are from one study typical of at least three generated under the same conditions. Means and standard errors are shown.

Fig. 3A illustrates that treatment with 7 and 10 mg Zn/L of nano-ZnO rapidly eliminated light output from the biosensor. Interestingly, lower nano-ZnO concentrations of 1.0 (Fig. 3A) and 5.0 (data not shown) increased light output above the control. With bulk ZnO no toxicity was observed up to 10 mg Zn/L, rather increased light output was observed (Fig. 3B). Treatment with 10 (not shown) and 1 mg Zn/L as the free ion (Fig. 3C) caused rapid loss in light output. The threshold value for reduction of Lux activity of the free ion was between 0.1 and 0.5 mg/L. As with the nano- and bulk ZnO, increased light output also was observed for the free ion, with activating concentrations observed between 0.1 and 0.05 mg/L of ionic Zn.

None of the treatments with Zn caused complete loss in culturability, rather they were bacteriostatic. Cells grew from the Zn-exposed samples at a delayed rate. Whereas colonies from the control cells could be counted in 2 days, those from cells exposed to zinc required at least 5 days. The culturability data shown in Fig. 3 are after 1 week of growth.

### Exposure to mixtures of nanoparticles

**Figure 4:**
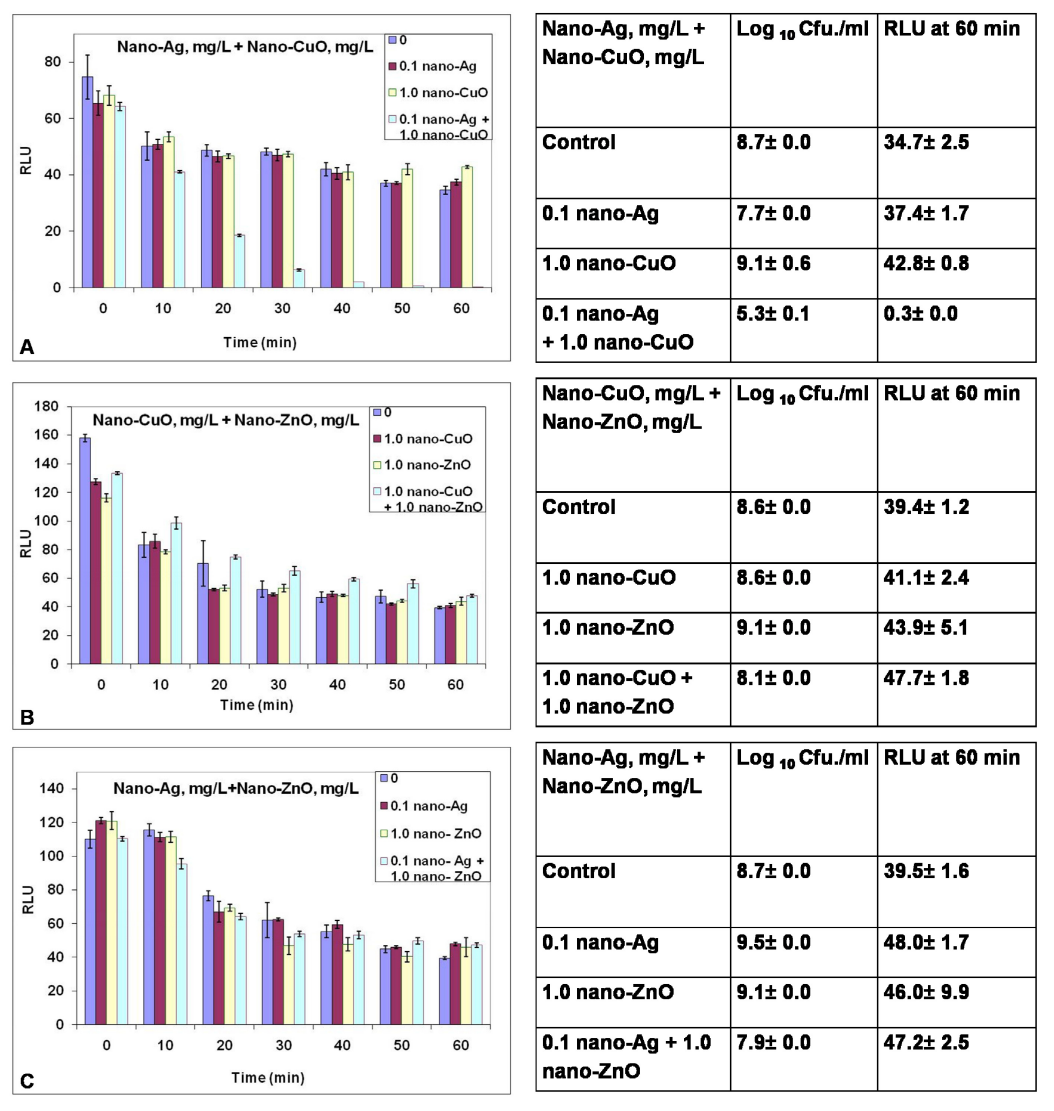
**Response of the *P putida *KT2440 biosensor to combinations of nano-Ag, nano-CuO and nano-ZnO at defined doses of mg Ag/L and mg Cu/L**. Changes in Lux output (Relative light units RLU) and cell culturability (colony forming units Cfu) are shown. Studies were performed as described in Methods. NCD = No culturable cells detected. Data are from one study typical of at least three generated under the same conditions. Means and standard errors are shown.

Fig. 4A demonstrates that co-treatment of the biosensor with 0.1 mg nano-Ag/L plus 1.0 mg/L of nano-CuO caused a time-dependent loss in light output whereas the individual NP treatments had no effect. The adjacent culturability data are after 1 week of growth on the plate medium – the longer time was required as the combined treatment caused bacteriostasis. Cell culturability declined but was not reduced to zero upon exposure to this NP mixture. NP interaction promoting loss in Lux output was not observed in cotreatments with 1.0 mg/L nano-CuO plus 1.0 mg/L nano-ZnO or 0.1 mg nano-Ag/L plus 1.0 mg/L nano-ZnO (Fig. 4B and 4C).

### Size determination of particles in aqueous suspensions of nano-CuO and nano-ZnO

**Figure 5:**
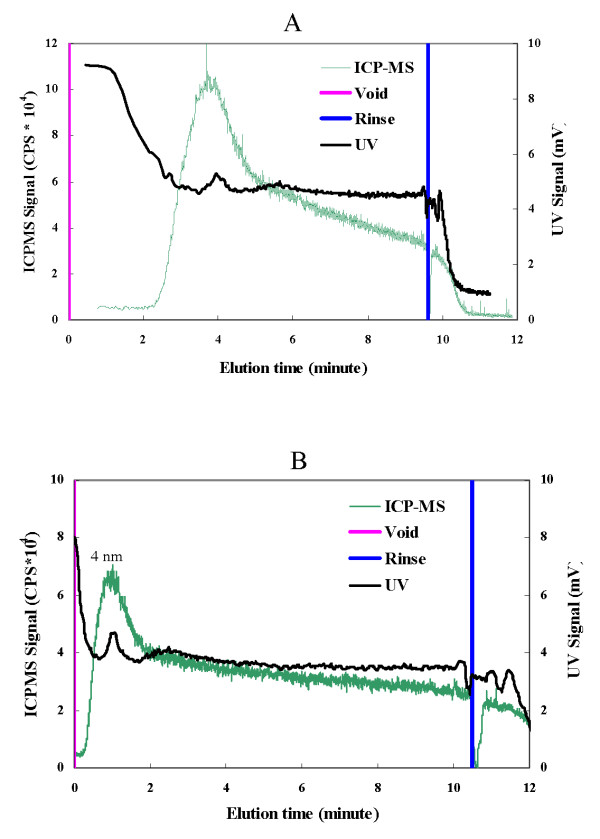
**Fractogram of materials remaining on the filter of a 450 nm filter as assessed by ICP-MS and UV signal for nano-CuO (A) and nano-ZnO (B) sampled under FlFFF condition II (Table 1), elution time was adjusted by the void peak**.

**Figure 6:**
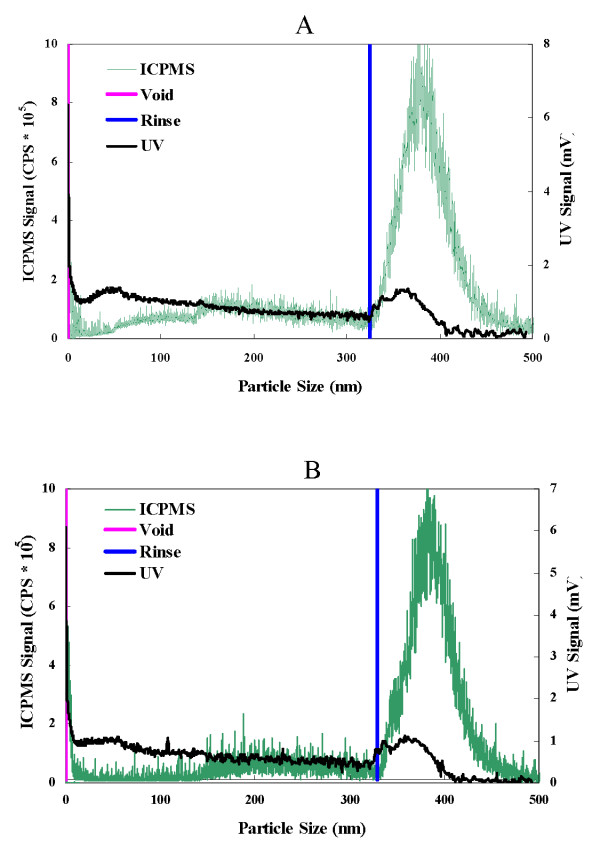
**Fractogram of materials present in the 450 nm filtrate of nano-CuO (A) and nano-ZnO (B) as assessed by ICP-MS and UV signal under FlFFF condition I (Table 1)**. Elution particle size was adjusted by the void peak.

FlFFF of the CuO and ZnO NP filtrates obtained using filters with a 450 nm cut off showed the presence of structures smaller than 5 nm (Fig. 5A and 5B). The material remaining on the filter surface for both nano-CuO and -ZnO (Fig 6A and 6B) contained poly-dispersed particulates in a broad size range from 70 nm to larger than 300 nm, with the bulk being larger than 300 nm, as demonstrated by the high ICP-MS counts during the rinse peak.

### Biosensor response to filtrates containing 5–200 nm particles from nano-CuO

Treatment of the biosensor cells with the filtrates passing through both 450 nm and 200 nm filters from a suspension of nano-CuO at 10,000 mg Cu/L caused dose dependent loss in light output. No toxicity was observed with a 100-fold dilution of the filtrate but no light was emitted when cells were treated with the × 10 diluted filtrate. The loss in Lux activity with the 10-fold diluted filtrate correlated with loss in culturability. Similarly, treatment with the filtrate prepared from 10,000 mg Zn/L from nano-ZnO at × 5 and × 10, but not × 100 dilution, caused partial loss in light output. In contrast to the filtrate from the nano-CuO, no change in culturability was observed for nano-ZnO (data not shown).

## Discussion

The biosensor constructed in the environmental isolate *P. putida *KT2440 effectively and rapidly, within minutes, demonstrated dose-dependent toxicity of NP of Ag, CuO and ZnO. These findings illustrate that the toxicity was not restricted to bacteria with pathogenic potential. Rather an environmental isolate, studied because of its bioremediation potential, was affected. The NP of Ag, CuO and ZnO were more toxic, causing loss of Lux activity in the biosensor, than their equivalent bulk materials indicating that the nano-size of the material was important. The findings that nano-Ag, nano-CuO and nano-ZnO reduced Lux activity were consistent with the observations by other groups that these NP caused bacterial membrane damage [6,10,13]. We speculate that such damage altered the membrane potential of the cell and, we presume, the availability of the FMNH_2 _required for the Lux activity. Consequently, Lux activity declined in the biosensor cells.

With Ag, the toxic doses of the NP and the ion were similar (~0.2 mg Ag/L) in the KT2440 cells. For Cu, complete loss of light output required exposure to 10 mg Cu/L from nano-CuO compared with 1.0 mg Cu/L of the Cu ions. Similarly, 7–10 mg Zn/L was required for toxicity of nano-ZnO compared to about a ten fold lower dose of Zn ions. Using nano-CuO and nano-ZnO from sources different from our own, nano-ZnO was more toxic than nano-CuO for *Vibrio fischeri *[17], compared with similar toxicity with for KT2440. Combinations of nano-Ag and nano-ZnO or nano-CuO and nano-ZnO were not interactive. However, the combination of nano-Ag plus nano-CuO was more inhibitory than their effects alone and the decrease in Lux correlated with reduction in culturability. These findings suggest that the target sites for nano-Ag and nano-CuO differed.

Toxicity as assessed with the pseudomonad biosensor was at lower NP levels than observed in other assays where culturability on solid or liquid media was the bioassay. For instance, in assays in rich medium, nano-ZnO toxicity required 126 mg Zn/L with *S. aureus *[18] and for *E. coli *and *B. subtilis *70 mg/L for nano-Ag [5] compared with 7–10 mg Zn/L from nano-ZnO and 0.3 mg Ag/L for the pseudomonad. The KT2440 bioassays were performed under conditions with no other added metal ions, thus, limiting possible competition with the heavy metal for bacterial binding sites. Likewise, the inorganic and organic materials that compose most bacterial growth media were not present. Such materials might otherwise complex the metals and change bioavailability.

Size and, thus, aggregation of the NP are important in nanotoxicity. For nano-ZnO, particles of 8 nm in size were more toxic to *S. aureus *than those that were reported to be larger (50–70 nm); these latter products were from the same Sigma-Aldrich source that we used [18]. Thus, it is interesting that we observed by FlFFF that 5 nm NP were present in the nano-CuO and-ZnO preparations. Exposing the biosensor to filtrates of nano-CuO and ZnO that would contain such particles showed dose dependent effects on light output and cell culturability. The FlFFF fractograms also showed that the aqueous NP suspensions prepared from manufactured NP powders were aggregated into poly-dispersed particulates ranging in size range from 70 nm to larger than 300 nm, with the majority of the Cu and Zn mass being associated with the larger particles.

Unlike the treatments with Cu or Ag, nonlethal doses of zinc from bulk, nano-ZnO and the ion increased light output above the control in the bioassays. To explore whether this was due to Zn activation of the promoter of the PP_0588 locus, we added zinc to a biosensor prepared with the fusion of the same *luxAB-npt *cassette to the promoter of the pseudomonad catalase gene. No increase in light output was observed with addition of Zn in this construct where the promoter region lacked a metal-sensitive motif (data not shown). These findings suggest that increased Lux activity with the KT2440 biosensor by Zn was promoter-driven, in agreement with the existence of a heavy metal-sensitive element in the promoter of the PP_0588 used in biosensor construct. Also, in the biosensor KT2240 strain we observed zinc caused bacteriostasis. Two other studies report that nano-ZnO was bacteriostatic to *Streptococcus *and *Staphylococcus *isolates in both broth medium or on solid agar plates [18,42]. Additionally the antimicrobial effect of nano-ZnO was reported to be sensitive to activation by the UV-radiation from laboratory lighting [18], conditions under which our assays were performed. Other studies on toxicity of nano-ZnO to mammalian cells found that solubilization of nano-ZnO as well as release of Zn ions from the NP contributed to activity [43].

Our observations confirmed that the biosensor generated with Lux as the output signal was a sentinel for cellular toxicity. Similar bacterially-based biosensors have been used previously to examine the toxicity of Cu and Zn in sludges [44]. Collectively, our findings show that NP preparations containing the heavy metals Ag, Cu and Zn were toxic to the beneficial environmental microbe, *P. putida *KT2440, suggesting that the NP at certain concentrations (≤ 1 mg Ag/L, ≈ 10 mg Cu, Zn/L) can be an environmental risk. The impact of the nano-metal oxides on cell culturability was dependent on the chemistry of the particles, with Zn causing bacteriostasis whereas Cu and Ag were bactericidal. FlFFF of the aqueous suspensions of the nano-metal oxides showed most of the mass was in aggregates greater than 300 nm although these ranged downward with another peak at 5 nm. Our findings suggest that further studies on determining the factors that affect aggregation of commercial NP in the environment are required. It is likely that such aggregation would reduce the deleterious effect of as-made NP on nontarget microbes. Implementing conditions promoting NP aggregation could alleviate point-source contamination.

## Competing interests

The authors declare that they have no competing interests.

## Authors' contributions

Experimental design and final analysis were conducted by AJA, WPG, and DWB. PG, AJA and BP carried out the Lux experiments. WH and WPJ carried out the FlFFF experiments. All authors contributed to writing the manuscript and have approved the final manuscript.
